# Impact of a multifaceted education program on implementing a pediatric palliative care guideline: a pilot study

**DOI:** 10.1186/s12909-015-0478-z

**Published:** 2015-11-02

**Authors:** Charissa Thari Jagt - van Kampen, Leontien C. M. Kremer, A. A. Eduard Verhagen, Antoinette Y. N. Schouten - van Meeteren

**Affiliations:** 1 Emma Children’s Hospital, Academic Medical Centre, Pediatric Oncology F8 Zuid, Meibergdreef 9, 1105 AZ Amsterdam, The Netherlands; 2 Universitair Medisch Centrum Groningen, Beatrix Kinderziekenhuis (code CA 72), Postbus 30.001, 9700 RB Groningen, The Netherlands

**Keywords:** Pediatric palliative care, Clinical practice guideline, Guideline implementation, E-learning

## Abstract

**Background:**

A national clinical practice guideline for pediatric palliative care was published in 2013. So far there are only few reports available on whether an educational program fosters compliance with such a guideline implementation. We aimed to test the effect of the education program on actual compliance as well as documentation of compliance to the guideline.

**Methods:**

We performed a prospective study with pre- and post-intervention evaluation on compliance to the guideline of the nurse specialists of a pediatric palliative care team for case management at a children’s university hospital. Eleven quality indicators were selected from 192 recommendations from the pediatric palliative care guideline, based on frequency, measurability and relevance. The multifaceted education program included e-learning and an interactive educational meeting. Four e-learning modules addressed 19 patient cases on symptoms, diagnostics and treatment, and a chart-documentation exercise. During the interactive educational meeting patient cases were discussed on how to use the guideline. Documentation of compliance to the guideline in the web-based patient-charts as well as actual compliance to the guideline through weekly web-based parent reports was measured before and after completion of the e-learning.

**Results:**

Eleven quality indicators were selected. The educational program did not result in significant improvement in compliance for any of these indicators. The indicators “treatment of nausea”, “pain medications two steps ahead” and “pain medication for 48 h present”, measured through parent reports, scored a compliance beyond 80 % before and after e-learning. The remaining indicators measuring compliance, as well as six indicators measuring documentation by chart review, showed a compliance below 80 % before and after e-learning.

**Conclusions:**

The multifaceted education program did not lead to improvement in documentation of compliance to the guideline. Parent reported outcome revealed better performance and might be the more adequate assessment tool for future studies.

## Background

Although life-shortening disease is rare during childhood, yearly about 4200 children in the Netherlands are entitled to palliative care [1]. Pediatric palliative care has recently gained interest within pediatrics and the World Health Organization (WHO) introduced a pediatric palliative care definition [2]. The Dutch Association of Pediatrics (NVK) has developed an interdisciplinary clinical practice guideline (CPG) palliative care for children in summer 2013 based on available evidence as well as expert opinions [3]. The main focus of this Dutch CPG is symptom management, decision-making and organization of care. The recommendations in the guideline aim at reducing variability of care, minimize under- and over-utilization of resources, and ultimately have the potential to improve the quality of palliative care [4]. So far, no formal implementation program was launched to promote its use. It is known that CPGs don’t implement themselves, and a well-organized implementation strategy is necessary to increase the guideline’s general performance and its effectiveness to change clinician’s behavior [5, 6]. Examples of successful strategies include multifaceted educational programs [7–9]. E-learning is reported to offer high flexibility, accessibility, satisfaction and cost-effectiveness [10–13]. Additionally, improvement of knowledge after e-learning when compared to non-intervention and other teaching interventions is reported [14, 15].

Our university children’s hospital has initiated a specialized pediatric palliative care team (PPCT) in June 2012 which consists, among other specialists, of five specialized pediatric nurse specialists that fulfill the role of case manager for children with life-shortening disease and their families. The team’s goal is to provide support from the moment the child’s disease is considered incurable until death has occurred. The nurse specialists organize, coordinate and support the care from primary, secondary and tertiary healthcare professionals and also provide aftercare [16]. The team’s clinical practice and advises in treatment should ideally be based on the Dutch CPG. Although this guideline was available on the internet directly after publication and a printed version was within the office of our PPCT, it is unknown to what extend the PPCT really used and followed the guideline.

We hypothesized that an education program could increase the compliance of the nurse specialists from the PPCT to the Dutch CPG. Our first research question was: what is the baseline compliance of the nurse specialists to the guideline, measured with selected quality indicators. Our second research question was: can introduction of a multifaceted education program, which we specifically designed for this purpose, improve the team’s compliance to the CGP.

## Methods

### Development of quality indicators

To allow measurement we aimed to use preset quality indicators, measuring compliance of the CPG. An extensive literature search did not reveal such indicators [17]. Therefore, in order to measure the adherence to the Dutch CPG, we developed healthcare quality indicators. All 192 recommendations mentioned in the CPG for the diagnosis, treatment and evaluation of symptoms in the palliative phase were considered as possible quality indicators. The four authors reviewed all 192 recommendations in a joint session and together selected all recommendations with a potential to be used as a quality indicator. The four authors then individually ranked ten of the selected 28 recommendations as possible indicators according to three criteria: measurability, incidence and its clinical relevance. The most frequent and highest ranked recommendations considering measurability and importance were selected to form quality indicators. All recommendations that were ranked within the top-ten of at least three authors were automatically selected. Recommendations ranked by two authors were selected if the mean ranking position was no higher than 5.

### The multifaceted education program

**Fig. 1:**
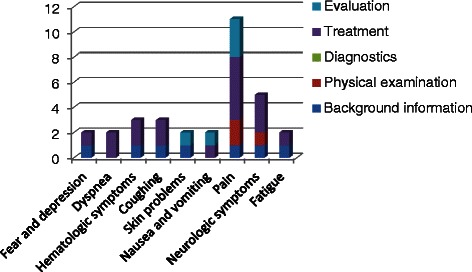
Distribution of subjects of e-learning questions. Figure 1 displays the distribution of the symptoms as addressed in the e-learning questions

E-learning modules and an interactive educational meeting were developed by two authors (CJ and AS) and were offered as a multifaceted education program for the nurse specialists of the PPCT. The goal of the e-learning modules is not to learn recommendations by head specifically, but primarily to stimulate regular review of the CPG and thus get familiar with using the CPG. The e-learning has four modules describing a total of 19 cases of patients with any life-shortening disease. Each module consists of 8–10 questions, addressing theoretical background as well as recommendations on symptom management given by the CPG. All chapters describing a specific symptom-group are addressed in at least one of the modules as is presented in Fig. 1. Since pain is frequently seen in palliative care, this symptom was addressed in all four modules. Each of the quality indicators is addressed in least once in one of the four modules. However, since the goal of the e-learning is to stimulate use and review of the complete CPG, the questions are not limited to the quality indicators.

During the e-learning session an open access available guideline was provided, stimulating the nurse specialists to find the answer in the CPG and thus get familiar with the use of it. Since the guideline stresses the importance of correct and complete documentation of symptoms and interventions, every module ends with a documentation assignment testing performance of complete documentation by the nurse specialist/participant. After completion of the module, direct electronic feedback is provided to the nurse specialist individually, with the correct answers as well as directions on which chapter and page the answer is displayed in the guideline and the exact text of the guideline is provided.

The different modules were offered to the nurse specialists for four consecutive weeks. All five nurse specialists participated in the e-learning. One week after the last module was offered and completed, the education program was completed with an interactive educational meeting with examples of actual recent patient cases and discussions on how and when to use the guideline.

Evaluation of effects of the education program as perceived by the nurse specialist was obtained 3 months after completion, through a short web-based questionnaire addressing experiences with the education program as well as self-evaluation of degree of current adherence to the guideline. Attendants approved that data could be used for research purposes.

### Assessment

Documentation of compliance as well as actual compliance to the CPG was measured during two periods; pre-intervention from August first 2013 until March 8^th^ 2014 and post-intervention from April 8^th^ until October 8^th^ 2014. The web-based patient charts, used for daily documentation by the PPCT, were reviewed to measure documentation of compliance. The charts were reviewed on occurrence of a clinical situation in which one of the recommendations used for the quality indicators would apply. All relevant reports were scored on whether compliance to the CPG was documented. Information on actual compliance to the CPG was retrieved from a web-based parent report on symptom occurrence and management. Parents were asked whether a symptom was present, and if so, the quality indicators concerning the symptom were literally asked to parents (Table 1). Neither the nurse specialists, nor the parents were informed that their reports would be assessed for this study. The Medical Ethical Review Committee (METC) of our university hospital the Academic Medical Centre in Amsterdam, considered the retrospective chart approach of our study to be within the regulations of the Dutch Medical Research Involving Human Subjects Act, with no requirement to retrieve informed consent from parents.

**Table 1:** ᅟ

Indicator	Chart review/ parental questionnaire	Ranking		
		Times ranked	Sum rankings	Mean ranking
1. Was pain documented with a VAS score	Chart review	4	8	2
2. Was nausea documented with a VAS score	Chart review	4	8	2
3. Was dyspnea documented with a VAS score	Chart review	4	8	2
4. Was treatment of pain evaluated with a change in VAS score	Chart review	2	2	1
5. Was treatment of nausea evaluated with a change in VAS score	Chart review	2	2	1
6. Was treatment of dyspnea evaluated with a change in VAS score	Chart review	2	2	1
7. Was nausea treated with either 5-HT3-receptor antagonist/D2-receptor antagonist/H1&AChm receptor antagonist	Parental questionnaire	3	14	4.7
8. Were the next two prescriptions to treat pain known to parents	Parental questionnaire	3	11	3.7
9. Was enough pain medication present to treat the pain for 48 h	Parental questionnaire	3	11	3.7
10. Was the patient with fatigue advised to keep a diary	Parental questionnaire	2	10	5
11. Was the patient with fatigue advised to spread activities	Parental questionnaire	2	10	5

The questions for parents were: Indicator 7: Did your child suffer from nausea? If yes please check the boxes of the medication that your child received. Indicator 8: Was your child in pain? If yes; do you know what the next two steps in pain medication will be? Indicator 9: Was your child in pain? If yes; do you have enough pain medication in house to treat your child for the next 48 h? Indicator 10: Was your child tired? If yes; were you advised to keep a diary? Indicator 11: Was your child tired? If yes; were you advised to spread activities through the day?

The ranking should be interpreted as follows: the first column describes how many of the four authors have rated this indicator in his or her top ten. The second column gives the sum of the positions of the rankings. The third column gives the mean ranking calculated as column 2 divided by column 1. The lower the score in column 2 and three, the higher was the indicator ranked

### Data analysis

We describe compliance to the CPG as well as documentation of use of the CPG, measured by predefined indicators, before and after the education program. If compliance was found in 80 % of the measurements for each indicator, it was considered as sufficient use of the CPG. We chose to test whether a compliance of 80 % was achieved, since this is the percentage we aimed to reach for adequate performance of care. Two different hypotheses were tested with the statistical program SPSS 20. First, we hypothesized that the multifaceted education program would result in a significant increase in use of the guideline. The difference between the before and after measurement of each indicator was assessed with a chi-square test. A chi-square test with a p-values of <0.05 was defined as a significant difference. Second, we hypothesized that after completion of the multifaceted education program, the nurse specialists of the PPCT would comply to the CPG in at least 80 % of the measurements of each quality indicator, as assessed with the z-test for single proportions.

A power-calculation deciding the minimum number of measurements needed to demonstrate an improvement from the pre-intervention adherence of 10 % to a post-intervention adherence of 80 %, requiring at least 13 measurements both pre- and post- intervention.

## Results

### Quality indicators

From the 28 selected recommendations on symptom management, 11 quality indicators relating to the recommendations in the CPG for pain, dyspnea, nausea, and fatigue were selected (Table 1).

### The multifaceted education program

**Fig. 2:**
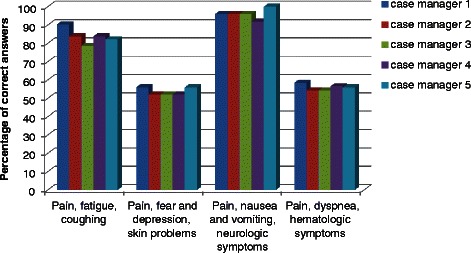
Displays the results of the nurse specialists on each of the four e-learning modules, shown as percentage of correct answers

The percentage of correct answers on the four e-learning modules per nurse specialist is shown in Fig. 2. The results are similar between the nurse specialists. During the interactive educational meeting, all participants showed a great deal of enthusiasm and eagerness to keep expanding knowledge and applicability of the guideline. All nurse specialists showed increased awareness of the importance and added value of correct use of the CPG, and stated to comply to the CPG increasingly. Evaluation of the education program by the nurse specialists revealed a mean satisfaction score of 7.6 out of 10 for the usefulness of the education program. The nurse specialists also rated their perceived improvement of compliance to the guideline after completion of the education program. For compliance to the guideline regarding evaluation and treatment of symptoms, their self-assessment mean score was 7.8 out of 10. The self-assessment mean score for documentation was 6.4 (Table 2).

**Table 2:** Results of evaluation of the multifaceted education program and its influence

	Mean score	Range
How do you rate the educational value of the multifaceted education program	7.6	6-8
After completing the education program I think I follow the guideline sufficiently regarding evaluation of symptoms	7.8	5-10
After completing the education program I think I follow the guideline sufficiently regarding treatment of symptoms	7.8	5-10
After completing the education program I think I follow the guideline sufficiently regarding documentation of symptoms	6.6	3-8
After completing the education program I am aware to document symptoms according to the guideline	6.4	4-9

Provides the scores of some of the questions of the web based evaluation form of satisfaction as well as perceived self-assessment by the nurse specialists. The questions were addressed as “to what extend do you feel…….”, and could be answered with a VAS of 1–10 for the degree of agreement, with 1 being the lowest score and 10 the highest

### Adherence to the guideline before and after the education program

We measured compliance to CPG and correct documentation of use of the CPG before and after the intervention. During the first evaluation period, 62 patients received support from the PPCT, of whom 22 patients had a malignant disease (MD) and 40 patients had a non-malignant disease (NMD). During the second evaluation period, 56 children received support from the PPCT of whom 19 patients had a MD and 37 patients had a NMD. Forty-five patients, who were supported by the PPCT during the first study period, were still supported by the PPCT at the start of the second study period. Eleven patients were newly introduced to the PPCT in between the two periods or during the second study period and thus included in the second test-period only.

Table 3 shows the number of patients with a clinical situation in which one of the recommendations used for the quality indicators could apply (identified by chart review or parent questionnaire), the number of measurements for each indicator, and the compliance for each indicator. There was no significant improvement in compliance to the CPG for any of the indicators after the education program. Before and after the education program, compliance to the CPG of at least 80 % of the measurements was seen in three quality indicators, “treatment of nausea”, “pain medications two steps ahead” and “pain medication for 48 h present”. More specifically these three indicators were all measured through parent reports (numbered 7–9). Although compliance and/or documentation of compliance was seen in some measurements of the other quality indicators, none of the other quality indicators showed a compliance in 80 % of the measurements. In one of the indicators, “evaluation of nausea”, no measurements could be performed after the education program due to absence of the symptom addressed.

**Table 3:** Results of the pre- and post- intervention measurements of performance of quality indicators

Indicator	Pre-intervention measurement			Post-intervention Measurement			Significant difference between pre- and post- intervention (chi-square)
	Measurements	Compliance to CPG Measurements N, (%)	Compliance >80 %	Measurements N / Patients N	Compliance to CPG Measurements N, (%)	Compliance >80 % (single test for proportions)	
	N / Patients N		(single test for proportions)				
1. Documentation VAS of pain (CR)	58 / 17	6, (10 %)	No (*p* < 0.001)	57 /12	6, (11 %)	No (*p* < 0.001)	No (*p* = 0.975)
2. Documentation VAS of nausea (CR)	9 / 6	0, (0 %)	No (*p* < 0.001)	11 /6	1, (9 %)	No (*p* < 0.001)	No (*p* = 0.353)
3. Documentation VAS of dyspnea (CR)	19 /8	0, (0 %)	No (*p* < 0.001)	21 /8	0, (0 %)	No (*p* < 0.001)	No
4. Evaluation with VAS of pain (CR)	17 /8	4, (24 %)	No (*p* < 0.001)	7 /5	0, (0 %)	No (*p* < 0.001)	No
5. Evaluation with VAS of nausea (CR)	1 /1	0, (0 %)	No (*p* < 0.001)	0 /0	0, (0 %)	-	No
6. Evaluation with VAS of dyspnea (CR)	4 /3	0, (0 %)	No (*p* < 0.001)	1 /1	0, (0 %)	-	No
7. Treatment of nausea (PQ)	5 /4	5, (100 %)	**Yes** (*P* = 0.87)	16 /2	15, (94 %)	**Yes** (*P* = 0.99)	No (*p* = 0.567)
8. Pain medication 2 steps ahead (PQ)	27 /9	22, (100 %)	**Yes** (*P* = 0.99)	32 /16	25, (78 %)	**Yes** (*P* = 0.39)	No (*p* = 0.750)
9. Pain medication 48h present (PQ)	26 /8	26, (100 %)	**Yes** (*P* = 0.99)	33 /17	30, (91 %)	**Yes** (*P* = 0.99)	No (*p* = 0.115)
10. Advised use of diary (fatigue) (PQ)	45 /18	0, (0 %)	No (*p* < 0.001)	55 /23	0, (0 %)	No (*p* < 0.001)	No
11. Advised spread of activities (fatigue) (PQ)	43 /16	0, (0 %)	No (*p* < 0.001)	54 /22	1, (2 %)	No (*p* < 0.001)	No

CR chart review, PQ parent questionnaire

Describes the compliance to the CPG for all eleven indicators before and after the multifaceted education program. The second and fifth column describe the number of available measurements for each indicator as well as the number of patients that were available for measurement. The third and sixth column describes the number of measurements which showed correct use of the CPG as well as the percentage of the total number of measurements. The fourth and seventh column describes whether a compliance of at least 80 % is seen for each indicator, assessed with a single test for proportions. The bold figures highlight the indicators where the 80% compliance was met. The last column shows whether a significant improvement in compliance is seen between the two measurements

## Discussion

We report a pilot study to investigate methods for improvement in compliance to the CPG for palliative care for children, by introducing a multifaceted education program. In contradiction to our hypothesis that the multifaceted education program would lead to a significant increase in compliance to the CPG, no significant difference is seen for any of the selected quality indicators before and after the education program.

Although Grol and Grimshaw found that structured implementation can improve adherence to CPGs [7], many reports argue that there is no magic bullet to change clinicians behavior to comply to a CPG [6, 18–20]. Weaver identified in an integrative review several barriers that can limit the success of implementation of palliative care processes. Barriers that could also be relevant in our setting are lack of qualified support services, lack of knowledge, lack of communication with medical setting, perceived lack of time, discomfort of physician, provider misconceptions, and finally a lack of comprehensive care culture [21]. Accordingly, recent studies on compliance to different pediatric guidelines, report compliance scores varying from 21 %, 43 % and 88 %, implying low compliance scores are regularly observed [22–24].

A multifaceted intervention including a combination of small group interactive postgraduate training, with personalized feedback, and additional instruction material, such as a website, was found to be more successful for implementation of a guideline than a single-faceted intervention [7–9]. One of the fastest growing education methods is e-learning which has been described as a dynamic, innovative and a rich way to provide learning opportunities [25]. Reported pros of e-learning are flexibility, accessibility, satisfaction and cost-effectiveness [10–12]. Cook and colleagues have shown that e-learning can increase students’ own control over the content, place and time of learning, and help students to gain knowledge and skills faster than traditional instructor-led methods [13, 15]. In addition, they compared e-learning interventions to other types of computer based educational interventions, and showed that interactivity, practice exercises, repetition, and feedback improved knowledge outcomes while using e-learning interventions [14]. So far, there are no reports that can replicate the positive effect of e-learning methods specifically on guideline implementation [26–30]. In our cohort, even after introduction of a multifaceted education program we could not show a significant difference between the pre-and post- intervention measurements.

We showed correct use of the CPG, defined as compliance in at least 80 % of the measurements, in three out of eleven indicators (numbered 7–9) before and after the intervention. These indicators evaluate the right choice of medicament to treat nausea, the upfront defining of pain treatment, and the upfront prescription of pain medication.

Two indicators (10–11), measuring the recommendations of the CPG to give certain advices to patients with fatigue showed low scores before and after the intervention. The low compliance scores on indicator 10 and 11 might be biased by the relatively short test periods of the study. Assessment of given advises to patients with fatigue, can be performed during the whole period of fatigue of the child. The PPCT might have provided specific advises according to the CPG at some point earlier outside our measurements periods. Since it is not prescribed and unwanted to repeat these advises on a weekly base no manifest application of the CGP could have been observed in our measurement episodes.

The scores in the indicators measuring documentation in the medical charts of compliance to the CPG (1–6) were low before and after the intervention. Although the low compliance scores may be in line with other publications reporting limited compliance [22, 24], several reasons may be suggested to explain the low estimated compliance in our study. During the interactive educational meeting as well as in the web-based evaluation of the education program, the nurse specialists expressed increased awareness as well as perceived improvement of compliance to the CPG. However, self-assessment of documentation of use of the CPG also revealed low scores. This might imply that the PPCT does comply to the CPG correctly but does not document the use as such and thus assessment of results through chart study might be an inadequate reflection of clinical practice. Due to the trans mural nature of our PPCT, the coordinating tasks, as well as the highly experienced nurses in the team, we did presume awareness of the importance of secure and complete documentation. However, reports describe that poor documentation of the performed tasks by nurses might lead to under-reportage [31, 32]. Paans et al., have screened patient records of 341 patients of 10 hospitals in the Netherlands to quantify the accuracy of different aspects of record documentation. The results show moderate accuracy described as 38 % for record structure, 20 % for admission data, 76 % of nursing diagnosis, 95 % for nursing interventions, 37 % for progress and outcome evaluations and 3 % for legibility of nursing reports [33]. Moreover, Abbaszadeh et al. performed a comparative study on the effect of e-learning compared to conventional training methods on nurses documentation [34]. Similar effects of an individual e-learning course and a conventional training method on nursing documentation imply that e-learning is not a superior education method to improve nurse’s documentation.

A strength of our study is that we were able to collect many measurements, by assessing web-based charts as well as parent reports of all patients receiving support of the PPCT. The easy and complete access will have limited the risk of information and selection bias. Moreover, we have developed a multifaceted education program addressing a national CPG, with high satisfaction and perceived effectiveness scores from participants that can easily be distributed to all professionals providing support in the palliative phase of children. Evaluating compliance to the CPG shortly after introduction, allows rapid implementation efforts to be developed, hopefully leading to effective implementation of the CPG and thus improving quality of care. A limitation of the study could be that not for all the indicators enough measurements were available. However, according to our power-calculation, for most of the indicators on documentation enough measurements were available for reliable analysis.

Assessment of charts has the limitation that only documentation of compliance is measured. This, together with the short test periods of the study, might have led to lower scores. Another interpretation could be that our education program was nog good enough. Perhaps lessons from the literature should have been taken more into account [21]. Finally although all members of the palliative care team were included in this study another limitation is the small number of nurses participating in the study.

Based on our study results we would argue that further research is needed to identify whether the low compliance scores are underestimated due to short study periods or suboptimal assessment, or the intervention was not optimal. Future research should focus on parent- or observer- based reports for adequate assessment of compliance and effectiveness of the multifaceted education program.

## Conclusions

In conclusion, in this study we showed that a multifaceted education program, including e-learning did not increase (documentation of) compliance to the CPG. Documentation of compliance, as measured by 6 indicators through web-based chart review, remained low after the multifaceted education program. Sufficient compliance to the CPG was seen in three indicators measured through parent reported performance before and after the intervention. Additional research is required to define different methods for assessment of guideline compliance in clinical practice as well as to investigate optimal education for guideline performance.

## Abbreviations

CPG: Clinical Practice Guideline
MD: Malignant Disease
NMD: Non- Malignant Disease
NVK: Dutch Association of Pediatrics (Nederlandse Vereniging voor Kindergeneeskunde)
PPCT: Pediatric Palliative Care Team
WHO: World Health Organization
